# Mycobacterial Biofilm: Mechanisms, Clinical Problems, and Treatments

**DOI:** 10.3390/ijms25147771

**Published:** 2024-07-16

**Authors:** Xining Liu, Junxing Hu, Wenzhen Wang, Hanyu Yang, Erning Tao, Yufang Ma, Shanshan Sha

**Affiliations:** 1 Department of Biochemistry and Molecular Biology, Dalian Medical University, Dalian 116044, China; xining_liu2002@163.com (X.L.); junxinghu2022@163.com (J.H.); 17889736446@163.com (W.W.); 18404789287@163.com (E.T.); 2 The Queen’s University of Belfast Joint College, China Medical University, Shenyang 110122, China; vip508006647@163.com

**Keywords:** biofilm, *Mycobacterium tuberculosis*, mycobacterial biofilm, drug resistance, antibiofilm

## Abstract

Tuberculosis (TB) remains a threat to human health worldwide. *Mycobacterium tuberculosis* (Mtb) and other nontuberculous mycobacteria (NTM) can form biofilms, and in vitro and animal experiments have shown that biofilms cause serious drug resistance and mycobacterial persistence. Deeper investigations into the mechanisms of mycobacterial biofilm formation and, consequently, the exploration of appropriate antibiofilm treatments to improve the efficiency of current anti-TB drugs will be useful for curing TB. In this review, the genes and molecules that have been recently reported to be involved in mycobacterial biofilm development, such as ABC transporter, Pks1, PpiB, GroEL1, MprB, (p)ppGpp, poly(P), and c-di-GMP, are summarized. Biofilm-induced clinical problems, including biofilm-related infections and enhanced virulence, as well as their possible mechanisms, are also discussed in detail. Moreover, we also illustrate newly synthesized anti-TB agents that target mycobacterial biofilm, as well as some assistant methods with high efficiency in reducing biofilms in hosts, such as the use of nanoparticles.

## 1. Introduction

In the past century, scientists have conducted a great amount of research on mycobacteria, including *Mycobacterium tuberculosis* (Mtb) and nontuberculous mycobacteria (NTM), such as *Mycobacterium smegmatis*, *Mycobacterium chimaera*, and *Mycobacterium avium*, but the concept of mycobacterial biofilm was only developed within the past few decades. The phenomenon of an aggregation of mycobacteria, observed in early years, was referred to as ‘pellicle’, ‘film’, ‘aggregates’, etc. [1,2]. These were the early conceptions of mycobacterial biofilm. However, little research has investigated the substances surrounding bacterial cells. In 1978, Costerton and colleagues claimed that bacteria use glycocalyx to mediate adhesion to targeted surfaces, both organic and inorganic [3]. They further elaborated on the tendency of the anchored single bacterium to proliferate and go through cell division to generate sister cells, thus forming a microcolony encapsulated with glycocalyx, in which some residue made of dead cells can also be observed. Surprisingly, they also found that this kind of coated pathogen was protected from being washed out by urine in the urinary tract. These findings inspired researchers to study the molecular components and biological functions of bacterial biofilm further.

In recent decades, a series of studies have proved that mycobacteria form biofilm both in vitro and in vivo [4,5,6]. A consensus has been reached, stating that biofilm, including mycobacterial biofilm, refers to a multicellular structure attached to a surface, with microbes embedded in an extracellular polymeric substance (EPS), comprising polysaccharides, proteins, and DNA produced by bacteria [7,8]. In Mtb, it was found that biofilm formation contributes to Mtb virulence and drug tolerance [4,6]. In 2022, tuberculosis (TB) remained a threat to human health, infecting 10.6 million people and causing 1.3 million fatalities worldwide [9]. Continuously emerging multidrug-resistant TB (MDR-TB) and extensively drug-resistant TB (XDR-TB) are the biggest barriers to anti-TB therapies. A deeper understanding and more research on mycobacterial biofilm are still necessary and will be helpful for the development of new anti-TB drugs that target biofilm, as well as novel therapies combining current first-line anti-TB drugs and antibiofilm agents. In this review, we will discuss the latest findings on mycobacterial biofilm, including the genes and clues involved in mycobacterial biofilm formation, the clinical problems caused by mycobacterial biofilm, and treatments with the potential to reduce mycobacterial biofilm.

## 2. The Components of Mycobacterial Biofilm

In biofilm, bacterial cells are surrounded by a particular environment, i.e., EPS, which not only contributes to the integrity of microorganisms but also serves as a physical barrier against threats [10]. The bacterial mass is just a tiny part of the dry mass of biofilm, whereas EPS accounts for more than 90% of its weight [11]. The components of EPS differ significantly among bacterial strains [11], and those of Mtb are polysaccharides, proteins, DNA, and lipids.

### 2.1. Polysaccharides

Trivedi and colleagues found a layer of polysaccharides at the base of microcolonies, thereby deducing that polysaccharides are the primary components of Mtb biofilms, serving as a bridge between microcolonies and substrata [12]. Using calcofluor white, they further confirmed that cellulose, which is produced by the bacteria dwelling in the biofilm, is the key polysaccharide in Mtb biofilm EPS. It connects bacteria and recruits the planktonic cells in the shape of micro-filaments [12]. Cellulose was verified to be a crucial component of the biofilm by the startling decrease in biofilm formation in cellulase-treated cultures [12].

### 2.2. Proteins

Using protein-specific FilmTracer SYPRO Ruby Biofilm Matrix Stain, Trivedi and colleagues presumed that protein is also an important component of Mtb biofilm EPS [12]. Lectin is a kind of protein secreted by bacteria and possesses high carbohydrate ligand specificity, thus playing an important role in bacterial adhesion and contributing to biofilm formation [13]. In 1989, Kundu and colleagues managed to isolate lectin from *M. smegmatis*, which was subsequently named ‘mycotin’. It can agglutinate erythrocytes and conduct cell aggregation, which can be blocked by mycobacterial arabinogalactan and yeast mannose [14]. Later, in 1994, Gosmiwa clarified the presence of mycotin-like molecules on the surface of Mtb and *M. avium*, proving their effect on the adherence of mycobacteria to macrophages [15]. However, distinct from *M. smegmatis*, the Mtb lectin mediates self-aggregation and is likely D-arabinose-specific instead of mannose-specific. This hypothesis was raised by Anton et al. based on their observation that adding mannose did not inhibit the aggregation of Mtb cells, whereas adding D-arabinose showed high efficiency in interfering with bacterial aggregation [16]. 

### 2.3. eDNA

In addition to polysaccharides and proteins, extracellular DNA (eDNA) has also been demonstrated to play an important role in bacterial aggregation and biofilm formation [17]. In mycobacteria, eDNA is detected mainly in slow-growing mycobacterial pathogens, whereas little eDNA can be found in rapid-growing ones [18,19]. Research on the conditions and genes involved in eDNA export has revealed that bicarbonate stimulates eDNA export in a pH-independent manner in *M. avium*, *Mycobacterium abscessus*, and *Mycobacterium chelonae* [20]. Several genes, such as those that encode FtsK/SpoIIIE-like DNA-transporting pores and carbonic anhydrases, have been confirmed to be involved in eDNA export in *M. avium*. Destroying eDNA with DNase I in slow-growing mycobacteria, including Mtb, promotes the susceptibility of mycobacteria to isoniazid (INH) and amikacin [18]. Disrupting eDNA with humanized monoclonal antibodies—which specifically recognize DNABII (a DNA-binding protein that stabilizes eDNA in NTM)—also makes the bacteria more sensitive to amikacin and azithromycin [21]. The establishment of biofilm can be promoted by substances that lead eDNA from B-form to Z-form, but it is impeded by those that have the reverse function [22].

### 2.4. Mycolic Acid

Mycolic acid is another key component of mycobacterial biofilm. There are three different forms of mycolic acids found in Mtb—α-, methoxy-, and keto-mycolic acids—of which α-form is the most common [23]. Possessing cyclopropane rings, mycolic acids promote cell wall complex synthesis and thus protect Mtb from threats. The structural integrity of mycolic acids also has a significant impact on the virulence of Mtb [24]. Ojha et al. showed that the EPS of Mtb biofilm contains abundant free mycolic acids, which contribute to forming the structure of Mtb biofilm [4].

## 3. Genes and Molecules Involved in Mycobacterial Biofilm Development

Since biofilm confers high virulence and strong resistance to mycobacteria, it is important to determine the genes and molecules involved in its development. Illumina RNA-seq technology was used by Ma and colleagues to detect these relative genes, revealing that the expression levels of 437 biofilm cell genes were different from their counterparts in planktonic cells. Specifically, 284 genes were upregulated, while 153 genes were downregulated. These results were also validated by qRT-PCR detections [25]. Hedge divided 115 biofilm- and quorum-sensing-associated proteins (BQAPs) into seven functional categories [26]. Here, we will discuss some of them in detail. Their functions and mechanisms in mycobacterial biofilm development are summarized in Table 1.

### 3.1. ABC Transporter

The ATP-binding cassette (ABC) transporters are a kind of protein that couple two processes, ATP hydrolysis and substance transport, and significantly influence bacterial physiology [35]. Ma et al. revealed that ABC transporter genes *Rv1217c* and *Rv1218c* had the most significant upregulation in biofilm cells compared with planktonic cells, as high as 9.2-fold and 10.6-fold, respectively. These two genes were verified to be associated with biofilm formation, and their expression could be suppressed by efflux pump inhibitors (EPIs), piperine, and 1-(1-naphthylmethyl)-piperazine (NMP) [25]. One of the mechanisms of ABC transporters that are involved in biofilm formation is the LpqY-Sug ABC transporter, which can transport mycolic acids outside of the bacteria [26,35,36]. Furthermore, other ABC transporters, including Rv1273 [37] and Rv3270 [38], act as multidrug efflux pumps and also contribute to mycobacterial biofilm formation.

### 3.2. Pks1

Using the transposon technique, Pang’s team screened out five Mtb mutants that had an impaired ability to produce biofilm. The worst mutant had defects in the *Rv2946c* (*pks1*) gene, and the other mutants were interrupted in *Rv0021c*, *Rv0199*, *Rv0252* (*nirB*), and *Rv3883c* (*mycP1*) [27]. Researchers later found that the *pks1* mutant can only bear immature and flimsy biofilms. However, when *pks1* is expressed at different genome sites in this mutant, more mature biofilm can be observed even compared with wild-type Mtb [27]. Furthermore, according to Ramos and colleagues, several genes participate in regulating the expression of *pks1*, of which *Rv0042c*, *sigK*, *Rv2258c*, and *Rv3557c* show a positive impact and *sigB*, *Rv2745c*, and *Rv3583c* regulate the expression negatively [39].

### 3.3. Peptidyl-Prolyl Isomerase-B (PpiB)

Peptidyl-prolyl isomerase (Ppiase) is a kind of cyclophilin that can help intercellular proteins fold, and it is widely expressed in human and bacteria cells. Two kinds of Ppiase exist in Mtb, PpiA and PpiB, which are encoded by *Rv0009* and *Rv2582*, respectively [40]. PpiB is relevant to mycobacterial biofilm formation [28]. Kumar et al. constructed *M. smegmatis* recombinant strains overexpressing Mtb PpiA (Ms_PpiA) and PpiB (Ms_PpiB) and found increased biofilm formation in Ms_PpiB, whereas Ms_PpiA and the control strain generated only basal level of biofilm [28]. Surprisingly, this feature of PpiB provides the possibility to interfere with Mtb infections. When two FDA-approved drugs, cyclosporine-A and acarbose, were added to an Mtb culture medium separately, biofilm formation significantly decreased. The blocking effects of cyclosporine-A and acarbose were accomplished by mitigating PpiB protein activity, and their binding sites in PpiB are conserved across infectious pathogens [28]. Furthermore, the presence of these two drugs increased the sensitivity of Mtb to the current anti-TB drugs INH and ethambutol, thus reducing their dosage in anti-TB therapy [28]. 

### 3.4. GroEL1

GroELs (or Cpn60s) are a group of chaperonins that help newly translated proteins fold and maintain specific structures [41]. Mycobacteria express two forms of GroEL—GroEL1 and GroEL2—through nonadjacent genes [42]. GroEL1 possesses a histidine-rich C terminus, and the attB site and has been verified to be important to mycobacteria biofilm maturity by research on two aspects. Firstly, integrating *M. smegmatis* phage Bxb1 at the attB site inhibits the function of GroEL1 and impairs the ability of *M. smegmatis* to form biofilm [29,42,43,44]. Secondly, the *M. smegmatis* mutant lacking the *groEL1* gene (Δ*groEL1*) fails to construct mature biofilm. Specifically, Ojha et al. clarified that GroEL1 is crucial for biofilm maturation instead of the initial attachment because GroEL1 modulates the synthesis of mycolic acids that are vital to biofilm maturation [29]. Though Mtb can encode similar GroEL1 proteins, it is still unclear whether the Mtb Δ*groEL1* mutant can exhibit similar defects in biofilm formation. Zeng et al. showed that Δ*groEL1* in slow-growing *M. bovis* BCG generates immature biofilm, indicating that GroEL1 plays a crucial role in the biofilm formation in *M. bovis* [30]. Furthermore, GroEL1 can counteract Cu^2+^ ions, which inhibits mycobacterial biofilm growth [45]. These studies show that GroEL1 has a significant impact on mycobacterial biofilm formation.

### 3.5. MprB

External molecules also influence mycobacterial biofilm formation. One of the human stress hormones—epinephrine—not only accelerates Mtb growth but also stimulates the establishment of biofilm. Lei and colleagues found that the biofilm induced by epinephrine shows better tightness and smoothness compared with vehicle-treated ones [31]. The *mprB* gene knock-down Mtb strain (*mprBKD*) displays slower growth, which indicates that the function of epinephrine is likely achieved by binding with MprB protein, the probable sensor of epinephrine [31].

### 3.6. Stringent Response-Related Molecules

The stringent response is a kind of response to nutrient starvation that exists ubiquitously in bacteria [46]. This process stimulates bacteria to divert nutrients from growth to survival and, ultimately, to form biofilm. Therefore, the genes and molecules relative to stringent responses are responsible for regulating biofilm formation. In Mtb, the stringent response is mainly regulated by the second message signals, including (p)ppGpp [47].

#### 3.6.1. (P)ppGpp

PpGpp and pppGpp, collectively referred to as (p)ppGpp, are pyrophosphate esterified to the 3’ carbon of ribose. Mtb possesses a gene named *rel_Mtb_* (*Rv2583c*), which reacts to starvation by generating intracellular (p)ppGpp [48]. Rel_Mtb_ catalyzes the transfer of pyrophosphate from ATP to GDP or GTP to synthesize ppGpp and pppGpp through its synthetase domain. Rel_Mtb_ also catalyzes the hydrolysis of (p)ppGpp into PPi and GDP or GTP via its hydrolase domain [48]. Weiss et al. observed that the mutation of the *rel_Mtb_* synthetase domain in Mtb resulted in a (p)ppGpp synthetase defect. Slow growth and delayed biofilm and pellicle formation were observed in this strain [32]. In short, stress and starvation stimulate the synthesis of Rel_Mtb_ to catalyze the production of (p)ppGpp, which performs a series of cellular activities including biofilm formation.

#### 3.6.2. Poly(P)

Inorganic polyphosphate (poly(P)) is another modulator of the stringent response [49]. Mtb expresses two polyphosphate kinases—PPK1 and PPK2—and two exopolyphosphatases—PPX1 (*Rv0496*) and PPX2 (*Rv1026*) [50]. These enzymes work collaboratively to maintain the homeostasis of poly(P). PPK1 promotes poly(P) synthesis, while PPK2, despite its name, predominantly exhibits the poly(P) hydrolysis function of PPX [50]. The balance of intracellular poly(P) is essential for not only Mtb cell survival during host infection but also biofilm formation in vitro [33,50]. Biofilm formation is absent or reduced in *ppx1* and *ppk2* mutants [33,49,50]. Therefore, it is conspicuous that both PPKs and PPXs interfere with biofilm formation, likely because they destroy the homeostasis of poly(P).

**Figure 1:**
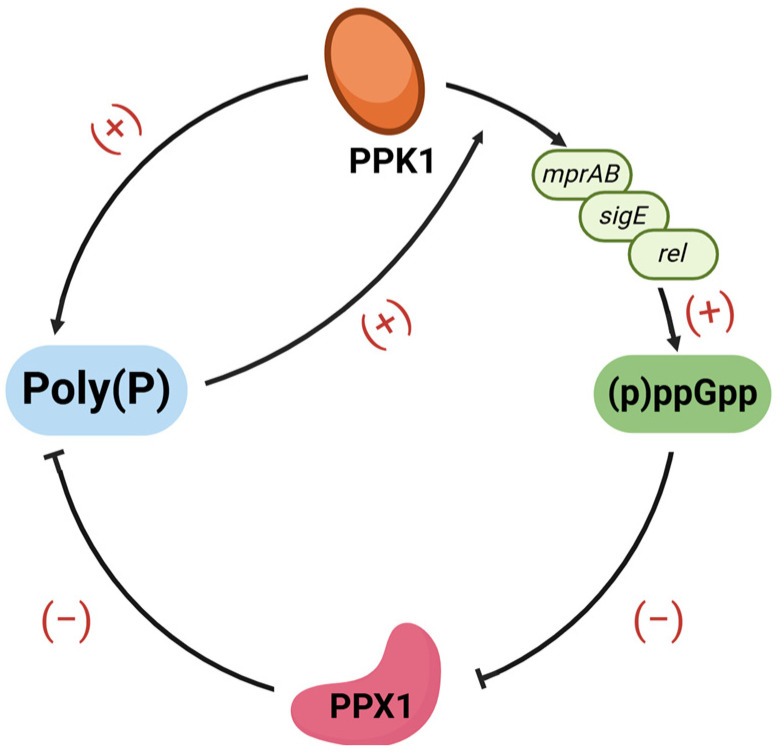
The interplay between poly (P) and (p)ppGpp. PPK1 promotes poly(P) synthesis through its polyphosphate kinase activity, while PPX1 hydrolyzes poly(P) with its exopolyphosphatase activity. PPK1 also promotes the production of (p)ppGpp by enhancing the transcription of *mprAB* and subsequently upregulating the expression of *sigE* and *rel*. The interplay between poly(P) and (p)ppGpp is reflected in two points. Firstly, Poly(P) enhances *mprAB-sigE-rel* signaling by phosphorylating MprA, thus upregulating the (p)ppGpp level. Secondly, (p)ppGpp displays an inhibiting effect on PPX1 and, consequently, promotes poly(P) accumulation. This figure was created with BioRender.com (accessed on 19 June 2024).

In addition, (p)ppGpp and poly(P) have strong interplay, as shown in Figure 1. Firstly, poly(P) has a positive impact on (p)ppGpp generation. Poly(P) can be used to phosphorylate MprA via PPK1 when facing ATP depletion; phosphorylated MprA then enhances *mprAB-sigE-rel* signaling and, consequently, promotes the production of (p)ppGpp [51]. Secondly, (p)ppGpp displays an inhibiting effect on Mtb PPX1 and, consequently, promotes poly(P) accumulation [52]. Therefore, (p)ppGpp and poly(P) are closely linked, together maintaining their level balance. 

### 3.7. c-di-GMP

Quorum sensing (QS) is a ‘cell to cell’ communication phenomenon. QS bacteria release ‘autoinducers’ (signal molecules) to regulate gene expression and alter a wide range of bacterial activities, including biofilm formation [53,54]. Mycobacteria possess a QS system that is regulated by cyclic-di-GMP (c-di-GMP)—a second messenger that exists extensively in bacteria [53]. C-di-GMP targets cellulose, polysaccharides intercellular adhesion (PIA), pili, and many other factors that can influence biofilm formation [55]. Intracellular c-di-GMP levels are closely associated with biofilm. High levels of c-di-GMP facilitate biofilm formation, while low intracellular c-di-GMP levels intensify biofilm dispersal [56]. A recent study conducted by Zhang et al. found a reduced level of bacterial metabolite gamma-aminobutyric acid (GABA) in biofilm cells during biofilm dispersal. The decrease in GABA promoted cellulase expression, disrupting the main EPS component cellulose, and this function was achieved by downregulating the c-di-GMP level [34]. Recently, Ling and colleagues clarified that c-di-GMP facilitates biofilm formation in *M. smegmatis* through the nucleoid-associated protein Lsr2, suggesting that Lsr2 acts as a c-di-GMP receptor linking the second messenger’s function to biofilm formation in mycobacteria [57]. 

Besides the above genes and molecules, certain high-centrality proteins and regulators, like RegX3, Rv0081, Rv0020c, Rv0097, and Rv1996, also participate in Mtb biofilm formation [26,58]. They are not isolated; instead, they establish complicated connections. In addition, a recent study on Mtb transcriptome indicates that non-coding RNAs (ncRNAs) are widely upregulated in Mtb biofilm and contribute to Mtb’s adaptation to biofilm growth [59]. Therefore, deeper investigations into these genes will contribute to our understanding of the mechanisms of mycobacterial biofilm development.

## 4. Clinical Problems

In persistent Mtb infections, mycobacteria exhibit reduced metabolic activity and slow replication owing to insufficient metabolic resources. These slow- or non-replicating mycobacteria can survive oxygen deprivation and form biofilms, which make them difficult to eradicate [60]. Biofilm-related infections differ significantly from those caused by planktonic microorganisms, showing more tolerance to immune responses and antibacterial treatment, and resulting in chronic and recurrent infections [61]. Furthermore, biofilm can act as ‘niduses’, shedding planktonic cells from time to time, making these diseases complicated to cure [62]. As a classical biofilm-forming mycobacteria, Mtb infects approximately one-quarter of the world’s population, of which 5%–10% develop TB [63]. Thus, to what extent does biofilm contribute to mycobacterial virulence?

### 4.1. Does Mycobacterial Biofilm Exist In Vivo?

The features of biofilm formed by mycobacteria have been described in detail; however, the existence of biofilm in vivo was verified only within the last few years. Researchers have illustrated several features of Mtb infections, such as prolonged treatment, immune escape, and a high recurrent rate, all of which resemble microbial biofilm infection [6]. In 2007, Lenaerts and colleagues infected guinea pigs with Mtb and found persistent bacteria at the acellular rim of primary granuloma shaped like microcolonies and clusters. They hypothesized that Mtb might have formed biofilm in vivo [64]. This hypothesis was further supported by the detection of mycolic acid and Mtb in the same area [65]. Recently, cellulose was used as a biomarker to identify the existence of biofilm in humans [66]. Cellulose is a crucial composition of Mtb biofilm but is absent in human cells [66]. Chakraborty and colleagues demonstrated Mtb biofilm formation in the lung tissues of infected mice and macaques using calcofluor white stain and cellulose-targeting fluorescence probe [6]. They further observed the existence of Mtb biofilm in the autopsied lung samples from humans infected with Mtb [6]. In addition, some mycobacterium strains isolated from patients can form biofilm in vitro [67,68]. These demonstrate the presence of Mtb biofilm in humans to a large extent, from which a series of clinical problems ensue.

### 4.2. Virulence

Biofilm is a microbial community with dynamic structures. After formation, biofilm does not remain static but ultimately enters a dispersal stage, in which cells are released to become planktonic [69]. Research shows that Cel6 (*Rv0062*) and Cel12 (*Rv1090*) are ubiquitous functional cellulose hydrolases in mycobacteria [70]. Chakraborty and colleagues created Mtb strains overexpressing cellulases Rv0062 and Rv1090, and reduced biofilm formation was observed in these strains. In mice, these strains failed to cause lung tissue damage but did maintain infection [6]. Another difference presented by these strains was a decrease in bacterial colony forming unit (CFU) after two weeks and four weeks of infection, indicating the role of biofilm in protecting bacteria from innate and adaptive immune responses [6]. When phagocytes are attracted by mycobacterial biofilm, they release antimicrobial oxidants, but these oxidants may fail to penetrate biofilm, thereby resulting in ‘frustrated phagocytosis’. Even worse, the phagocyte enzymes secreted by phagocytes may further cause significant damage to surrounding host tissues [71]. Moreover, our previous study revealed that Rv0062 has an important impact on biofilm dispersal based on its cellulase activity [34]. Rv0062 can hydrolyze the cellulose in the biofilm of *M. smegmatis*, which was responsible for the extensive spread of mycobacteria in mouse lungs [34].

### 4.3. Biofilm-Associated Infections

Some infections are closely and even directly associated with mycobacterial biofilm and many of them are caused by biomaterials. In Chinese and European cities, mycobacteria account for the largest proportion of bacteria that exist in urban air [72]. They also emerge in hospital water systems and some medical devices, which can lead to nosocomial infections [73,74]. Nearly half of hospital-acquired infections occur in patients who have received foreign body implantation, such as catheters, cardiac pacemakers, joint prostheses, and prosthetic heart valves, all of which are suitable for biofilm formation [75]. Many rapid-growing mycobacterium species, like *Mycobacterium fortuitum* and *M. chelonae*, are a menace to public health, colonizing and forming biofilm in intravascular catheters and consequently causing bloodstream infections. Removing the catheters is usually required to end the infections [74]. Yamamoto and colleagues used *M. avium*, *Mycobacterium intracellulare*, *M. abscessus*, and six different kinds of materials (polypropylene, acrylic, silicon, glass, titanium, and steel) to analyze NTM biofilm formation on medical devices. The results showed that these mycobacteria tend to form biofilm around air–liquid interface areas, and materials are factors that influence the amount of biofilm formation [76]. 

Another example of biofilm-related infections in hospitals is the infection of *M. chimaera* on heater–cooler units (HCUs). *M. chimaera* is a member of the *M. avium* complex, which grows slowly [77]. HCUs are vital for maintaining a patient’s blood temperature during cardiac operations. However, the water tank inside these devices may be contaminated by microorganisms like *M. chimaera* and its biofilms, making HCUs a place that produces and releases *M. chimaera* bioaerosols, which can reach surgical sites and the surrounding environment [78,79]. Measures have been proposed to decontaminate HCUs, but the existence of biofilm makes the process even more challenging [80]. Patients receiving cardiothoracic surgeries always suffer from an increased risk of *M. chimaera* infection [73], which has high mortality and requires reoperation to be eliminated [81].

### 4.4. Drug Resistance

According to the WHO Global Tuberculosis Report, tuberculosis is a worldwide threat, causing more than 1 million fatalities each year. However, the standard TB treatment achieves only 85% success [9]. Mtb evolves resistance to drugs, resulting in patients developing MDR-TB [82]. Although no clinical evidence supports that mycobacterial biofilm leads to treatment failure so far, biofilm-related drug resistance has been found in in vitro experiments and animals. It was found that biofilm cells do not grow better than planktonic cells, but they show increased survival [83,84]. Statistically, Ojha et al. found that approximately 10% of Mtb within a biofilm did not respond to INH even in a concentration beyond MIC; furthermore, high rifampicin exposure significantly attenuated Mtb biofilm viability in the first three days but failed to cause any damage in further treatment [4]. In another study, groups of mice infected with Mtb were given different therapies—INH and rifampicin oral treatment with or without nebulized cellulase. The results showed lower pulmonary tissue destruction and fewer live bacilli in the cellulase-treated group, indicating that biofilm can hinder the function of chemotherapeutic agents [6]. 

As a peculiar form of bacteria colony, biofilm has unique mechanisms for developing drug resistance. The normal mechanisms of drug resistance, such as efflux pumps, modifying enzymes, and target mutations [85], do not contribute significantly to biofilm drug recalcitrance. Even sensitive bacteria that are susceptible to antibiotics genetically become recalcitrant once they form biofilm [86]. These mechanisms are illustrated as follows: First, the surrounding EPS serves as a diffusion barrier against large molecules [84]. As for smaller molecules, the blocking function of biofilm can be seen as it deactivates them before they enter cells. Since EPS is negatively charged, positively charged agents, for example, aminoglycoside antibiotics, have retarded penetration because they are trapped by EPS [87]. Second, the efficiency of anti-TB drugs is challenged by the changing environment. Biofilm has microscale gradients comprising nutrients, wastes, and metabolic products that cooperatively influence its microenvironment [88]. A slight change in oxygen and pH, for example, can result in frustrated antibiotic activity [86]. Another important mechanism is that some cells in biofilm grow rather slowly or even stop growing due to nutrient limitation [71]. Because most of the current antimicrobials are designed to inhibit biosynthetic processes, they are ineffective at eliminating bacteria in a quiescent state, which are either slow-growing or dormant [87]. However, investigations of genetic mechanisms of drug resistance in biofilm focused mainly on *P. aeruginosa* and *Escherichia coli* so far; what happens in Mtb biofilm has not been clarified and needs to be explored.

## 5. Treatments 

### 5.1. Drugs Targeting Biofilm

In the 20th century, 12 to 13 chemical substances were validated as drugs that can fight TB, of which INH, rifampicin, ethambutol, and pyrazinamide are first-line drugs usually used in combination. For instance, the combination of INH and pyrazinamide or rifampicin possesses the highest potency [89]. Patients with TB are always required to receive regular treatments with first-line drugs for a long period. Short-course chemotherapy has also been used to treat drug-sensitive tuberculosis [89]. However, owing to Mtb’s waxy cell wall, slow growth rate, ability to form biofilm, and development of drug tolerance, clearing Mtb infections is challenging, requiring at least 6 months of treatment with traditional drugs [90]. Therefore, novel approaches and drugs with more efficiency, especially drugs that target Mtb biofilm, are urgently needed to cope with drug-resistant TB.

**Figure 2:**
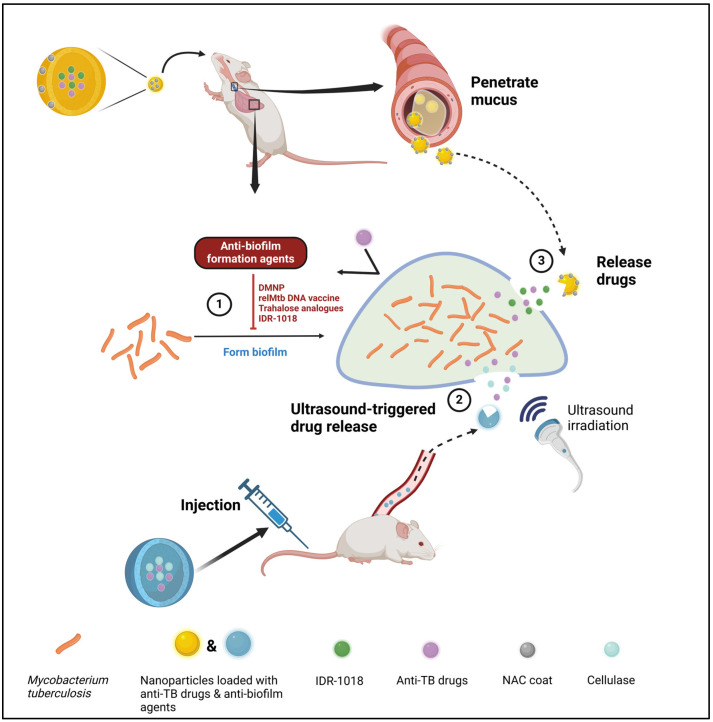
Anti-TB treatments in mice based on antibiofilm agents. Three strategies have been reported in current years. (1) Some antibiofilm formation agents, such as DMNP, trehalose analogs, and IDR1018, inhibit Mtb biofilm formation, enhancing the bactericidal activity of traditional anti-TB drugs. (2) Nanoparticles carrying IDR-1018 and traditional anti-TB drugs were coated with the mucus-penetrating agent NAC. After inhalation, it can penetrate host mucus via NAC and reach the infection points, releasing IDR-1018 and anti-TB drugs to prevent biofilm formation and kill planktonic Mtb cells. (3) Nanoparticles carrying cellulase and traditional anti-TB drugs were injected into host blood, which released cellulase and anti-TB drugs with the assistance of ultrasound irradiation. The formed mycobacterial biofilms were destroyed by cellulase, which improved the efficiency of anti-TB drugs. This figure was created with BioRender.com (accessed on 19 June 2024).

The recalcitrance of biofilm is phenotypic and reversible [83]; therefore, chemical substances that can either impede biofilm formation or destroy the formed biofilm can make a great contribution to TB treatment (Figure 2). Many features are required for an ideal antibiofilm agent. It should be able to kill bacterial cells rapidly to prevent them from transforming into biofilm phenotypes in advance, target slow-growing and non-growing cells, penetrate EPS or inhibit its generation, intervene in cell communication in biofilm, etc. [91]. However, it is nearly impossible to discover an agent with all of these features. It would be hopeful to find one that possesses some of these functions or to use them in combination. Here, we discuss a few newly developed antibiofilm agents in detail. Their functions and mechanisms are briefly summarized in Table 2.

#### 5.1.1. Intervening in Rel_Mtb_

As mentioned above, the stringent response is a crucial process relevant to biofilm, and Rel_Mtb_ is the main regulator of this process. Tkachenko and colleagues synthesized an analog of diterpene—4-(4,7-dimethyl-1,2,3,4-tetrahydronaphthalene-1-yl) pentanoic acid (DMNP) [92]—and found that this molecule possesses antibiofilm activity against *M. smegmatis*. They further verified that Rel_Msm_ is the target for DMNP to display this function. According to them, *rel_Msm_* gene expression and the mycobactericidal effect of DMNP increase simultaneously, and both *rel_Msm_* knock-out strains and a wild-type *M. smegmatis* strain exposed to DMNP exhibit atypical biofilm formation [92]. Therefore, this Rel_Mtb_-interfering agent has the potential to be used as a biofilm-eradicating drug to assist conventional antimicrobials against Mtb infections.

#### 5.1.2. Intervening in Trehalose Catalytic Shift

Trehalose is a glucose disaccharide in Mtb. It is a pivotal constituent of the cell wall components trehalose monomycolate (TMM) and trehalose dimycolate (TDM) [99]. As mentioned above, the former is needed to transport mycolic acid, a component of biofilm EPS in mycobacteria [26], while the latter can release free mycolic acid through enzymatic hydrolysis [100]. These facts indicate that trehalose plays a crucial role in biofilm formation. When facing hypoxia, there is a catalytic shift in trehalose metabolism, which deprives trehalose of TMM and TDM biosynthesis and utilizes it for central carbon metabolism (CCM) intermediate production instead. CCM promotes ATP and antioxidant production and, finally, contributes to drug tolerance. This process is closely associated with TreS, a trehalose synthase that catalyzes the conversion between trehalose and maltose [93]. Wolber and colleagues synthesized a series of trehalose analogs (including 2-, 5-, 6-TreAz) and proved their antibiofilm activity towards *M. smegmatis* after being transported into cells by the trehalose-specific LpqY-SugABC transporter [94]. Lee et al. ascribed this ability of trehalose analogs to their competitive inhibition of TreS, which has an identical architecture to trehalose [93]. Recently, they synthesized a panel of azidodeoxy and aminodeoxy-D-trehalose (TreAz and TreNH2) analogs and determined that 6-TreAz and 2-TreNH2 selectively inhibit biofilm formation in an LpqY-SugABC-dependent manner by interfering with the trehalose catalytic shift in Mtb [95]. All of these findings suggest that trehalose analogs can be regarded as a novel tool for inhibiting mycobacterial biofilm. 

#### 5.1.3. Promoting Biofilm Destruction

In addition to targeting biofilm formation, promoting biofilm degradation is an ideal strategy for fighting biofilm-forming bacteria. A better curative effect can be obtained when patients suffering from TB receive antimicrobials together with nebulized cellulase [6]. In one study, Zhang and colleagues used *M. bovis* BCG as an Mtb model to study the antibiofilm effects of cellulase. The results showed that after BCG strains cultivated with a mature biofilm were added via cellulase in different concentrations, the biofilm biomass value decreased in a concentration-dependent manner. Moreover, nanoparticles loaded with cellulase and levofloxacin showed better biofilm-eliminating and -dispersing effects compared with those loaded solely with levofloxacin [96]. 

#### 5.1.4. Multi-Function of Antibiofilm Agents

Occasionally, some antibiofilm agents were found when investigating the properties of specific chemicals. Although current drug delivery systems are useful therapeutic adjuncts in TB treatment, problems arise because of the limited surface area with which pores can carry only small amounts of drugs [101]. Metal–organic frameworks (MOFs) are an ideal delivery material, possessing large surfaces, pore volumes, and biocompatibility [102,103]. Kumar and colleagues elaborated on IITI-3, a Cu-based MOF, and encapsulated INH in it to create a novel form, INH@IITI-3. Interestingly, the results showed that INH@IITI-3 not only caused a prominent decrease in *M. smegmatis* and *M. bovis* but also interfered with *M. smegmatis* biofilm formation [97]. 

Antibiofilm substances may also possess other functions that contribute to TB treatment. For example, C10 is an agent with antibiofilm activity that also enhances the bactericidal effects of INH by blocking hypoxia-induced INH tolerance [104]. It was the most powerful Mtb biofilm inhibitor among the candidates selected by Flentie’s team [104]. Another example is antimicrobial peptides (AMPs), which comprise cationic and hydrophobic amino acids and display antibacterial functions by killing bacteria directly [83]. They are also potential antibiofilm tools and immune regulators; therefore, they are referred to by another term—host defense peptides (HDPs) [105]. HDPs precisely target the stringent response to display their antibiofilm property. For example, IDR-1018, a synthetic HDP, tends to bind (p)ppGpp preferentially and, therefore, exhausts (p)ppGpp from cells in vivo [98,105]. In addition, Yadav et al. found that human senescence marker protein 30 (huSMP30), a multifaceted protein consisting of various enzymatic and cellular functions, inhibits *M. smegmatis* biofilm formation due to its lactonase activity [106]. 

### 5.2. Methods to Assist Antibiofilm Agents

#### 5.2.1. Ultrasound-Triggered Nanoparticles

Nanoparticles have tiny structures and can carry small drugs and molecules to specific locations and release them at a controlled speed [107]. Poly (lactic-co-glycolic acid) (PLGA) is a widely used nanoparticle owing to its low toxicity [108]. Zhang et al. synthesized CL@LEV-NPs, a kind of nanoparticle containing cellulase (CL) and levofloxacin (LEV), with PLGA as the shell and CL and LEV constituting the core [96] (Figure 2). In their experiment, aside from CL@LEV-NPs, some groups of mice were treated with ultrasound (US) at a frequency of 42 kHz and an intensity of 0.34 W/cm^2^ for 5 min, while other groups were not. The results showed that the biofilm biomass was significantly reduced after receiving CL@LEV-NPs combined with US treatment. The BCG biofilm treated with US before applying CL@LEV-NPs showcased serious damage, with wrinkles and holes under a scanning electron microscope, while those without US presented relatively small injuries. Although the US treatment killed some bacteria, the CL@LEV-NPs + US group showed a significantly reduced mycobacterial load in mouse lungs compared with the group only treated with US. All the outcomes elucidated the potential effect of ultrasound-triggered nanoparticles in treating mycobacterial biofilm. 

#### 5.2.2. Nanoparticles with Mucus Penetrating Agents

Biofilm is not the only barrier that impedes the entry of antimicrobials. Human respiratory tracts are covered with a mucus layer containing water, proteins, lipids, salts, and cellular fragments, of which mucin is a major component [109]. The structure and thickness of mucus also influence drug delivery efficiency; they are distinctive in different anatomical positions, and the impact of mucus on drug delivery depends on these features, as well as age, disease, etc. [110]. The mucus makes it difficult for anti-TB agents to reach cells, thereby contributing to drug resistance and prolonged treatment [111]. 

A novel idea proposes that the function of anti-TB drugs can be enhanced when combined with antibiofilm agents and mucus-penetrating systems, and this has been tested on mice, as shown in Figure 2. N-acetylcysteine (NAC) is a mucolytic agent that also has antibiofilm properties [112]. Sharma and colleagues synthesized the IDR-1080 peptide, combining it with porous PLGA microspheres (PLGA-MS) with/without anti-TB drug INH, and these microspheres were then wrapped with NAC to confer these microspheres with the ability to thrust into the thick mucus, finally forming NAC-coated mucus-penetrating particles (NAC/PLGA-MPP) [111]. The researchers further compared the mobility of PLGA-MS and NAC/PLGA-MPP in mucus, as well as their anti-TB activity in vitro and in vivo. Compared with PLGA-MS, NAC/PLGA-MPP not only showed a better mucus-penetration ability but also caused a larger proportion of biofilm biomass reduction. In animal trials, NAC/PLGA-MPP has shown potent bactericidal properties and higher efficiency in reducing granuloma nodules and lesions compared to free IDR-1018 peptides and PLGA-MS. Therefore, inhaling NAC-coated polymeric particles—which have both anti-biofilm and mucus-penetrating properties—could be a potential adjunct to assist short-term therapies and help anti-TB agents reach inflammation sites. This might contribute to shortening treatments, lowering drug doses, and decreasing drug use frequency, thus enhancing the regularity of medication [111].

## 6. Conclusions and Perspectives

As a multicellular architecture surrounded by EPS, mycobacterial biofilm is composed of polysaccharides, proteins, lipids, and eDNA. Several genes and molecules play important roles in mycobacterial biofilm formation and are either relevant to the production of biofilm compositions or involved in inducing biofilm formation. The existence of biofilm in vivo was verified by using calcofluor white staining on cellulose and has posed serious clinical problems, including biofilm-related infections, powerful virulence, high recurrence rates, etc. Therefore, agents that can interfere with biofilm formation or promote biofilm destruction are urgently needed. However, traditional anti-TB drugs exhibit only a small impact on Mtb biofilms. Within the last decade, a variety of novel drugs and agents targeting mycobacterial biofilm have emerged. Most of them intervene with biofilm formation, and some can increase biofilm hydrolysis. When used as an adjunct therapy, these antibiofilm agents can eliminate biofilms, thus enhancing the potency of anti-TB drugs and mitigating inflammation. A few innovative agents have been created to assist antibiofilm drugs in penetrating the robust barrier of bacterial cells and arriving at certain locations. Thus, deeper investigations into the composition of biofilm and novel combination therapies are crucial for TB control.

However, the literature described in this review has several limitations. Firstly, most of the research mentioned in this review was conducted on Mtb models, like *M. smegmatis* and *M. bovis*. To ascertain the outcomes, similar experiments using Mtb specimens are required to prove the results. Secondly, in vivo clinical trials are also needed to bridge the gap and adequately use antibiofilm drugs in patients. Thirdly, other methods of using these antibiofilm agents remain to be investigated. For example, when a patient with TB needs a trachea cannula, drugs can be applied to the tube before intubation to prevent biofilm formation.

## Figures and Tables

**Table 1:** Genes and molecules involved in mycobacterial biofilm development.

Factors	Gene Function	Effect on Biofilm	Mechanism	Reference
LpqY-Sug-ABC transporter (*lpqY*, *sugA*, *subB*, *sugC*)	Trehalose transporter	Promote biofilm formation	Return trehalose to the bacteria, which assists the efflux of mycolic acids outside the bacteria	[26] (*M. tuberculosis*)
Pks1 (*Rv2946c*)	Polyketide synthase involved in lipid synthesis	Promote biofilm maturation	Not clarified	[27] (*M. tuberculosis*)
PpiB (*Rv2582*)	Peptidyl-prolyl cis-trans isomerase accelerating the folding of proteins	Promote biofilm formation	Not clarified	[28] (*M. tuberculosis*)
GroEL1 (*Rv3417c*)	60 kDa chaperonin 1 promoting the refolding of peptides	Promote biofilm maturation	Physically associated with FASII components to modulate the synthesis of mycolate and mycolic acid	[29,30] (*M. smegmatis* & *M. bovis*)
MprB (*Rv0982*)	Two component sensor kinase	Promote biofilm formation	Sensor of epinephrine	[31] (*M. smegmatis*)
(P)ppGpp	-	Promote biofilm formation	Signal of nutritional stress, regulated by Rel_Mtb_	[32] (*M. tuberculosis*)
Poly(P)	-	Promote biofilm formation	Modulate stringent response, homeostasis is required	[33] (*M. tuberculosis*)
Cyclic-di-GMP	-	Promote biofilm formation; associated with biofilm dispersal	Regulate quorum sensing	[34] (*M. smegmatis*)

**Table 2:** Antibiofilm agents.

Agents	Mechanism	Effect on Biofilm	Reference
DMNP	Target (p)ppGpp synthesizing protein Rel	Inhibit biofilm formation	[92] (*M. smegmatis*)
Trehalose analogues (TreAz, TreNH2)	Competitively inhibit TreS-mediated trehalose catalytic shift	Inhibit biofilm formation	[93,94,95] (*M. smegmatis* & *M. tuberculosis*)
Cellulase	Hydrolyze cellulose	Promote biofilm destruction	[6,96] (*M. tuberculosis* & *M. bovis*)
IITI-3	Not clarified	Inhibit biofilm formation	[97] (*M. smegmatis*)
IDR-1018	Bind and deplete (p)ppGpp	Inhibit biofilm formation, promote biofilm destruction	[98] (*P. aeruginosa*)
